# Unleashing the power of a willow bark‐derived tannin to alleviate behavioral deficits in a preclinical model of Alzheimer’s disease

**DOI:** 10.1002/alz.092849

**Published:** 2025-01-09

**Authors:** Ezaldean A Kahil, Anisha Jackson, Kruti Patel, Lori A Coward, Bernadette D'Souza, Gregory Gorman, Patricia Jumbo‐Lucioni

**Affiliations:** ^1^ Samford University McWhorter School of Pharmacy, Birmingham, AL USA; ^2^ University of Alabama at Birmingham, Birmingham, AL USA

## Abstract

**Background:**

Despite some advances in treatment, a cure for Alzheimer’s disease (AD) remains elusive. Disease hallmarks include heightened neuroinflammation and oxidative stress, associated with progressive decline in mobility and cognitive functions. Natural compounds provide a valuable reservoir of novel bioactive substances with therapeutic potential, fewer side effects, and increased affordability. The plant‐derived tannin 1,2,3,4,6‐Penta‐O‐Galloyl‐β‐D‐Glucose (β‐PGG) displays potent antioxidant, anti‐inflammatory, and neuroprotective properties *in vitro*, but *in vivo* evidence is limited. This study assesses the dose‐dependent efficacy and therapeutic mechanisms of β‐PGG in mitigating age‐dependent mobility deficits in a Drosophila melanogaster model of AD.

**Method:**

A fruit fly line overexpressing the human amyloid precursor protein (hAPP) and β‐site APP‐cleaving enzyme (hBACE), in neurons was used as AD model. Newly eclosed flies were supplemented with 0, 5, or 10µM β‐PGG and locomotion was assessed at 7, 14, 21, and 30 days via a negative geotaxis assay. The number of flies passing 2‐, 4‐, and 8 cm marks in 10 seconds was recorded by genotype, sex, age, and treatment. Oxidative stress sensitivity was measured by quantifying 48‐hour survival to paraquat exposure. Flies were homogenized to determine levels of β‐PGG metabolites by LC/MS/MS. Data were analyzed via a two‐way ANOVA to test the effects of genotype, treatment and their interaction in young (7‐ and 14‐days old) and old (21‐ and 30 days old) flies.

**Result:**

Young non‐supplemented AD females (p<0.0001) and males (p<0.0001) moved significantly slower than controls. β‐PGG significantly ameliorated locomotion deficits in young AD flies. AD females (p<0.0001) and AD males (p<0.0001) supplemented with 10µM β‐PGG were significantly less movement impaired than their non‐treated counterparts. Regardless of genotype, old females supplemented with 10µM β‐PGG were significantly faster than untreated cohorts (p<0.05). Conversely, old AD but not control males supplemented with 10µM β‐PGG were significantly less movement impaired than their untreated counterparts (p<0.0001). At 48 hours, 2‐week β‐PGG supplementation significantly improved survival to paraquat among control females but not in males (p<0.01).

**Conclusion:**

Our findings provide strong evidence that β‐PGG supplementation mitigates age‐associated mobility deficits in our preclinical model of AD. Benefits from this supplementation may delay physiological aging in a sex‐specific manner.

